# Water as a Remedy in Disease

**Published:** 1852-07

**Authors:** John C. Darby

**Affiliations:** Lexington, Ky.


### WESTERN JOURNAL

OF

### MEDICINE AND SURGERY.

JULY, 1852.

Art. I.—*Water as a Remedy in Disease.* By John C. Darby, M. D., of Lexington, Ky.

My attention was first called to the use of cold water in fevers, externally applied, by Dr. Cooke, Professor of the Theory and Practice of Medicine in Transylvania University, while I was a student in that institution. Dr. Cooke strongly recommended the practice taught by Dr. Currie. I have since several times read Dr. Cooke's Review of Currie's Medical Reports. At the same time we were very fully instructed by Dr. B. W. Dudley, as to the use of warm water as a local application or bath in almost all forms of inflammation. But I did not begin to prescribe the cold affusion or cold bath in fevers to any extent, till about the year 1845; when my attention was again particularly called to the subject by notices in newspapers of the *Hydropathic Practice.* I again read Currie, and such works on Hydropathy as I could obtain. In 1847, being in bad health, I went to New England to recruit, and to see that country, and took the opportunity to visit Brattleboro, Vermont, and examine into the water-cure system for myself. Dr. Wesselhoeft, the very gentlemanly proprietor, received me with great kindness, and treated me with marked courtesy. There were from seventy-five to eighty patients under treatment. I was introduced to nearly all of them, and as it was a rare thing for a "regular doctor" to visit such an establishment, except to find fault and to criticise, and as I gave them all every assurance that such was not my purpose, I had every opportunity afforded me for examination and investigation. I inquired particularly into the history of almost every case; and indeed the patients seemed to take pleasure in having me listen to their past experience as invalids. I passed the time very pleasantly, and learned a good deal; and shall always remember my sojourn of ten days at Brattleboro, as among the most pleasant of my life—I would say, in passing, that all invalids who have made up their minds to try this practice, will, in my opinion, be pleased with Brattleboro. Dr. Wesselhoeft is a man of good sense, well informed in his system; and I believe him to be an honest man and a gentleman; and have no doubt that he will always give a candid opinion to all who consult him. As to the establishment, I can hardly conceive of one being more thoroughly adapted to the purpose. There is an abundant supply of cold freestone water, and the various baths are all admirably arranged.

What I have said above, I feel to be due for the kindness and marked civility with which I was received and treated at Dr. Wesselhoeft's establishment.

I was not in any sense a patient for water treatment, and so informed Dr. Wesselhoeft. But as I went there for information, I asked him to allow me to try his various baths; which he readily consented to, and for which he made no charge—my object was to ascertain how far cold ablutions could be safely practiced *when the temperature of the body was not above the standard of health.* Dr. Currie had taught us how to use water in fevers. He says, "the affusion of cold water may be applied in a case of fever when there is no sense of chilliness present, when the heat of the surface is steadily above what is natural, and when there is no general or profuse perspiration." Dr. Currie, it seemed to me, had laid down and clearly established the rules to be observed in the use of cold water *in fevers*—I desired to study the other side of the question;—the use of cold affusions when there was no unnatural heat, and in other complaints besides fever.

I concluded that, if I would submit to the most powerful baths, so far as safety was concerned, they would include the less powerful. I was packed in the wet sheet three times; I took the running sits (from a cold spring) three times, and stood under the highest and largest douche twice. I found them all not only safe, but delightful and invigorating—I did not try the dripping sheet, nor did I apply a wet compress. I submitted while there to the regimen of the establishment, and at the expiration of the time (ten days) I found myself much improved in strength, and in a feeling of well-being.

It is unnecessary to give a description of the various forms of Hydropathic baths; it is to be presumed that nearly every reading man in the country understands what is meant by "packing in the wet sheet," "sits bath," "douche," "dripping sheet," &c. &c. The general conclusion to which I came as to the water-cure system was this; that it is admirably adapted to the treatment of many varieties of chronic disease, such as rheumatism, dyspepsia, piles, chronic bowel complaints, neuralgia, and that form of ill health which is the result of hard study or long continued and close confinement to business in cities; cases in which a close diagnosis would show that there was no particular organ diseased. I speak now of Hydropathy as practiced in an establishment; I will have something to say of some of the appliances of Preissnitz in common-out-of-door practice, after a while. I suppose that the cases of chronic diseases which, with the above view, properly come under the Hydropathic physician, must be treated in *an establishment.* To make it the system and practice of Priessnitz, various baths have to be prepared; requiring, in the first place, an inexhaustible supply of water; and the water must be cold, and soft or free-stone water; moreover, as other laws of Hygiene are made to form a part of the system, the establishment ought to be in the country, or in villages, where the patients can take their daily walks through pleasant groves, away from the noise, and bustle, and dust, and heat of a city.

The first effect of the treatment is decidedly tonic. So far as I could learn at Brattleboro, there was very rarely a patient who was not delighted with the practice for the first few weeks; because it made them feel stronger and more cheerful, with a better appetite and more refreshing sleep. But after a while they begin to be depressed; the treatment is taking a stronger and deeper hold upon them, and they now become discouraged. This condition lasts for a longer or shorter time in different individuals until what is called the crisis comes. This crisis consists in the breaking out upon some part of the body of a rash, or of boils and various forms of cutaneous eruption. The patients generally improve when these occur, or at any rate they feel better, except the annoyance from the boils or eruption. But a majority of the cases are not so fortunate as to be relieved when the crisis appears: they get better for a time, and then fall back again; and perhaps it may be many months before they are cured. My opinion is, that the practice, as it is commonly pursued in

Hydropathic establishments, i. e. to be packed in the wet sheet at day-light in the morning, with its accompaniments, sweating, plunge into a cold bath, &c.; then a sits bath about 10 or 11 o'clock, A. M., or a douche, or both; to be followed by another sits bath in the afternoon; and to wind up with a foot bath at night, is too much, to be continued for any great length of time; that in chronic cases, after it has been pursued for three or four weeks, or at farthest five or six weeks, there ought to be an intermission; that then the patient should be required to take the usual amount of exercise, and to live upon the prescribed regimen [which is always good enough] for a week or two; and that so far as water is concerned it should be limited to washing the body every morning, or the dripping sheet if you choose. Chronic diseases, to be treated upon the water-cure system, must be treated in an establishment especially fitted up for the purpose. Hydropathic doctors have an advantage over regular practitioners; their patients belong almost all to the better class of society; they are persons who have ample means, and who can afford to leave home and be absent for months at a time at a heavy expense. It is the interest of the Hydropathic doctors to have the patients remain at the establishment for as long a time as possible; and they seem to be justified in requiring them to remain until their health is fully restored; but if the patients would consult their own advantage, they would take a week or two's recreation during a season, and travel about, or visit some mineral spring, for change of scene and of society, if for nothing else.

The application of cold water as a daily bath may be used with comfort and pleasure through a long life-time; but to use it as a remedy in health, i. e. as it is prescribed for invalids, would be as injurious after a certain time as if a man were to take 20 grains of calomel every day.

Some impostor in Ohio, a few years ago, started the humbug of "tomato pills," as a substitute for calomel, and they were sold by a druggist in Lexington. I answered the imposition at the time by simply stating the fact, that tomatos were known to be a wholsome article of food was conclusive proof that they did not possess any of the qualities, powers, or properties of calomel; and so with the water cure system: as I remarked above in respect to one class of the patients who are benefitted by water treatment, they have no particular organ diseased; all that is the matter with them, is, that their whole system is relaxed, and worn down by excessive labor, either bodily or mental, and many of them would be as certainly cured by a tour over the western prairies, or a sojourn at the sea side, or at some of the mineral springs as at a water-cure establishment.

When a man starts a new system, either in education, in medicine, in religion, or any thing else, he is apt to forget how long the world has done without him and his system. Fortunately for the human family, all knowledge does not centre in the brain of one man; nor is it confined to any race or nation.

I am also satisfied that medicines ought not to be excluded from the treatment of patients in Hydropathic establishments. I have demonstrated to my own satisfaction, that water, as directed to be used as a remedy in disease by Currie and by the Hydropathists, is not incompatible with the use of medicines or *drugs* in the treatment of *acute disease.* This I will illustrate presently by giving some cases. And from what I saw at Brattleboro, and from the facts I have met with in Hydropathic books, I am certain that, many of their patients laboring under chronic affections would be much more speedily cured, if they were permitted to take some such medicine as a good physician would prescribe for them; and that some would be cured, who, as it is, are not cured at all.

The philosophy of health and of disease consists in part, in a knowledge of the influence of the physical agents, by which we are surrounded, upon man's animal being.

It is the province of the physician to understand those general laws, and to keep them in view when called to the bed-side of a patient. Little benefit can be expected from medicines proper, unless the physician is governed by these great laws. It is a truism, which must be admitted by all candid and thinking men, that whatever may fairly be said *to cure* a patient, (using the term in its common acceptation), must do so in accordance with the laws of the animal economy, whether the man who prescribes understands those laws or not. The allowances and qualifications to this principle are these: 1st. The patient might have got well without *the* remedy, or without *any* remedy. 2d. He may have recovered in spite of the disease and remedy together. But yet there are cases where there can be no doubt, that the individual's life was preserved or saved by the remedy. I will give one case, by way of illustration. Several years ago, I treated an old lady for (as I believe) inter susception. She had been vomiting for nearly four days; the matter thrown up appeared to be fecal; the pain was local and severe. When I first saw her, I gave an emetic, believing the disease to be colic; after that I gave nothing but opium in some form to relieve the pain and to quiet the stomach, and I used injections of warm water constantly and freely. But the patient grew regularly worse, and finally was almost exhausted. Happening, when I called during the fourth day, to have a galvanic battery in my buggy, I concluded to try it. It was applied by an individual, who held one of the knobs, placing the other hand over the bowels of the patient, and moving it about for fifteen or twenty minutes—it acted like a charm, the bowels acted promptly and freely, and the patient was entirely relieved, and she was soon in her usual health. Now I say that this patient would have died, in all reasonable probability, if the battery had not been used. The remedy must have acted in accordance with the laws of the animal economy, and yet I have no idea how it did act. I have selected the above case, because it does not properly come under the head of any particular system of medical practice. It illustrates the principle, for which I am contending. The fact that I have not been able to find out how the battery acted, does not affect the principle.

Well, it may be asked, am I writing to defend the Hydropathic practice? I answer that I am not. But I maintain, that the Hydropathic practice, as such, is worthy of the investigation of the regular faculty, and demands it. I maintain that it has developed and is constantly developing some important principles; and I believe that there are many cases, especially such as those referred to, such as have no special malady, but are worn down by excessive labor, mental or bodily, who ought to be recommended by their physicians to try the practice. The medical profession have perhaps benefitted the world as much by teaching mankind the laws of Hygiene, and the simple means of relieving disease in families, as they have ever been able to do at the bed-side. Whatever promises to preserve the health of man and to prolong his life, commends itself to the medical profession.

The practice of Currie had become almost obsolete when the practice of Preissnitz became known. The world is under many obligations to Preissnitz for reviving the practice of Currie, if for nothing else.

M. Louis says, in the conclusion of his volume upon fever, that bleeding and blistering appeared to have afforded more relief than any other remedies tried by him. I think that he gives the history of forty-seven cases that died. The most remarkable symptom, or one of the most remarkable and constant symptoms in these cases, was the heat of the skin. They were typhoid cases. Now, I

maintain that, had those patients been bathed according to the rules laid down by Currie, when their bodies were burning hot, M. Louis would not have had to express a doubt in his concluding remarks as to whether any remedy had been of any avail. What is fever? I answer that it is the generation of animal heat by the lungs so rapidly as to interfere with, or prevent the healthy performance of all the other functions of the body. It involves an increased action of the heart, but this is compatible with health; as when we take active exercise, or when the heart is excited by mental emotions. The chief element of fever is the continued, rapid generation of animal heat by the lungs; so as, among its first effects, to prevent the stomach from doing its office; it rejects food for the most part, or if the food is received, it is not well digested. There being then no supply of new matter to furnish fuel for this increased generation of heat, or combustion of oxygen; or to supply the waste of the body, the patient's body is necessarily reduced; and if there is a tendency to degeneration of the bowels, the glands and the mucous membrane become implicated. Now, I maintain that bathing the body with cold water, time and again, as may be necessary according to the rules of Currie; or the dripping sheet bath, according to Preissnitz, is a more rational and a more scientific practice than to bleed or to blister.

It is not necessary to discuss the *modus operandi* of the cold water practice in fever. To do justice to the question would require many pages. Suffice it to say, that the body is rapidly cooled; that the action of the heart is tranquillised; in a word, that, for the time being, the fever is broken, and the nervous system is so much invigorated, that the chances are always in favor of the fever's not returning; or if it does return, it will not be so high. Dr. Currie directs that the cold affusion should be resorted to as soon as possible, after the fever is fully developed; during the first hot stage if you can. Since 1847, I have treated many cases after this plan. But I also give medicines. I give calomel without any fear of salivation, and I have never seen a case where the gums were touched, in which I could say that it was in any way attributable to the water. I do not confine the practice to fever cases. Whenever called to a patient, whose skin is hotter than natural, with an excited pulse, and especially if the head be hot and painful, I either order the cold affusion; or that the patient be well sponged with cold water, let the disease be what it may. I rarely ever bleed in idiopathic fever; where it is complicated with inflammation I may bleed, or give an emetic; and there are cases in which I may not deem it necessary to use the cold water. The cold water practice of Currie, and even that of Preissnitz rationally understood, is not at all incompatible with the use of emetics, cathartics, or any of the ordinary methods of practice. Where there is an establishment with all the appliances of the water-cure system, I have no doubt that many cases of acute, as well as of chronic disease, may be successfully treated without medicines. But in ordinary practice, in town and country, where a physician may or may not have any convenience, he cannot, if he would, confine himself to water alone. Physicians need not fear that their practice will be injured by Hydropathy. If they will study Currie's Medical Reports, as old as they are, and study the Hydropathic practice, they will find themselves much better qualified to treat disease, than if they implicitly follow such authorities as Louis and Bouillaud. Too many lives are daily sacrificed to science "falsely so called." The study of anatomy, physiology and pathology can never be less important than they are at the present time; no discovery, however brilliant, can ever do away with a thorough practical acquaintance with the various branches of medical science; and it will always be a matter of the first importance to be able to make a correct diagnosis. But while all this is true, it still remains a fact, that the great principles of physiology are too much lost sight of; the influence of the physical agents by which we are always surrounded upon our bodies, in health and in disease, is too little attended to. Water is one of the most common, and one of the most useful of all those agents. It may be modified in its application almost indefinitely. Let the profession, then, return to it again and give it a fair trial; and it will turn out, I doubt not, as it did when Currie published the first edition of his work; from one end of the land to the other, there will come up the reports of physicians, testifying to its great value as a remedy in fevers and other diseases.

Case I. Shortly after my return from Brattleboro, in August, 1847, I was called to see a patient, whose malady was what I would call old fashioned bilious fever. He was a large athletic man between twenty-five and thirty years of age. The fever was very high; his skin excessively hot; his pulse strong, full, and frequent. I ordered him to be undressed, and then to lie down on the floor, and as there was a spring under his room, we had the water brought up bucketful after bucketful, until I had dashed over him some five or six. We then rubbed him dry quickly, and put him to bed. He immediately fell asleep and did well for some hours; but the fever rose the next day at the usual hour; and I again repeated the cold affusion; but as the floor of his room was covered with water, when I first bathed him, and as his wife was his only attendant and nurse, I made him go into the yard the next time, (his room was on the ground floor, and he had only to step out of the door to be in the yard), and then poured as many buckets as before over him. The fever was again removed for the time being; and again he went into a comfortable sleep. I bathed this man some five times in the same way on succeeding days, and had no occasion to repeat it afterwards. I gave such medicines as I thought suited his case; among which was calomel. He was not salivated, and by the end of ten days was entirely convalescent.

Case II. Soon after the father recovered, one of his sons was taken with fever, which seemed to be of the same character. I visited this boy, a child of seven or eight years of age, very seldom; for his mother, a very sensible woman, told me that she could manage his case herself;—that whenever the fever rose, she made him undress, and go into the yard, and lie down, where she poured three or four bucketsful of water from the spring over him, and then rubbed him dry and put him to bed. His fever did not last longer than that of his father. I state the case of the boy to show that when the practice has been once tried in a family, although there may be great opposition at first, it soon gives way, and the family become so much pleased with the remedy, that they have no hesitation in trying it on all occasions.

Case III. The next case in order, was a complicated case of acute rheumatism. I was called to see this man on the 16th of January, 1849. During this winter I saw nine cases of partial paralysis; it was for the most part, in my practice, complicated with rheumatism; and as I learned from other physicians that they had many cases of a similar nature, I concluded at the time that paralysis was then an epidemic. I lost no case so affected. The medicines used were such as I employed in the case of the man, a history of whose disease I am about to relate. He was a large and portly man, about fifty years of age; red hair, and thoracic temperament; a blacksmith by trade. He told me that he had been ill for a week; that he had had no physician. Some twenty years before he had had a severe attack of rheumatism, which had confined him to his bed several months; that on the present occasion he had not slept at all for a week, and could not use either arm or leg. He was paralysed. When I placed my fingers on his wrist to feel his pulse, he said it gave him great pain; that he could not bear to be touched. I found his pulse strong and full, his skin very hot, and his tongue coated. The patient was immediately placed in half a hogshead of cold water, and as it had been standing out of doors, it had some ice in it; so he was washed all over with ice-water. After rubbing him dry and putting him to bed, I bandaged each arm, first with a wide bandage wet with cold water, and then covered this with a dry bandage. That night he slept well. He was also given cathartic medicine, and put upon a solution of the nitrate of potash, (ʒj to the quart of water), as a drink. The next morning when I visited him, he informed me that he had again been bathed in the tub, but that when he got into the water he found it uncomfortable, the water being too warm; his friends fearing the effect of very cold water, had carefully removed all the ice, and filled the tub up with water just from the spring. He required them to go out and get some ice and snow, and put it into the water. The cold bathing was continued from day to day for about a week; he took such medicines as I thought were indicated, but I put more reliance upon the cold bathing, and the wet bandages around the arms than upon any thing else; I dismissed the case as entirely convalescent on the 28th. It was some weeks before he recovered his strength, but he had no backset, and became, in a month or two, as strong and well as ever, and now boasts of his recovery under the water practice.

Case IV. *A Case of Croup.*—Patient, a little boy between four and five years of age—I have never seen a more marked case of croup than this. I first tried to vomit the child with ipecacuan and tartar emetic, but effected nothing; the emetic medicine was rejected by the time it was swallowed, without relieving the patient in the least. I was called to see the child at 2 o'clock, A. M., and it was now 4; finding that my little patient was growing rapidly worse, I determined to try the wet compress. I wet a large towel in ice water, and folded it well over the chest and around the neck, and put a dry cloth over it. I also gave a decided dose of calomel. I then gave directions to renew the under wet cloth, as often as it might become hot. But before I thought it prudent to leave, the child had gone to sleep, and breathed with perfect ease. As this was during the prevalence of cholera in Lexington, and as I was very busy, I did not see the patient again until 9 o'clock, A. M. I found him sitting at a little table, eating broiled chicken and bread. He said he was well; which was the fact. There was no further difficulty. The child was well. The same treatment was used under my advice this summer, the case was croupy catarrh; and the child, a boy of seven or eight years of age, had been ill for four days. The remedy was altogether successful. The mother of the child was very much opposed to the application of the wet towel, but was much gratified at the result.

Case V. July 11th, 1849, I was called to see a young man about twenty-five years of age, who had the cholera. I found him cold; his body and limbs felt cold, and he complained very much of feeling cold. His skin was moist, but the perspiration was not excessive, nor did it ever become so. The discharges were rice water. They were not very large, though frequent. I gave him brandy, mixed with ice water, freely. He took a dose of calomel every four hours, until the discharges became bilious, and after that, the interval between the doses was sometimes increased. But I could not by any means bring about a re-action. I tried various stimulants, besides external applications, but all to no purpose. He continued in this condition for three days and nights. On the morning of the fourth day, I determined to try the cold dash. He was laid upon a sheet spread on the floor, and I then poured nearly a barrel of fresh cold water over him. He was then quickly rubbed dry and placed between blankets in bed. Re-action commenced in a very short time, and we had no farther difficulty. He was very slightly salivated. This young gentleman has enjoyed better health since he had the cholera than he had done before. I consider it all-important, when cold water is used by affusion, that there be assistants upon whom reliance can be placed, to rub the patient dry, and to do it quickly. The brisk rubbing is absolutely necessary when the patient is cold.

I have given six cases, in three of which the temperature of the body was above the standard of health. In the croup case the temperature of the body was not above the healthy standard; in the case of croupy catarrh there was a slight fever. I have not specified particularly the medicines used in these cases. I do not consider it necessary, because my intention is merely to show, that cold water may be safely used in certain cases; and that it does not interfere with the use of medicines, but assists them; that calomel may be given with as much safety when we are using cold water, as if it was not employed at all. As I have already remarked, I consider the practice recommended by Dr. Currie particularly adapted to the fevers of the south and west; I mean miasmatic fevers, intermittent and remittent, where the re-action is excessive. I rely upon the cold affusion almost altogether to subdue the fevers of children. I prefer it to bleeding or emetics. I have the child undressed, quickly washed all over, and then wiped dry, and put to bed. I also frequently require my patients to wear a wet compress. I have afforded great relief in cases where there was a painful state of the bowels, by applying a wet compress over the bowels, with a dry one over the wet one, after the fashion of the hydropathists. The patients wear these compresses continually for weeks, day and night. The under cloth has to be re-wet whenever it becomes too hot, or begins to get dry. The outer cloth is necessary to restrain evaporation, and to prevent the clothes of the patient from becoming damp. In one case of asthma, this wet compress has proved of very great service. The patient was a single lady, about thirty-five years of age; she had been afflicted with asthma from childhood; had tried a great many remedies with little or no relief; the attacks were frequent and very severe. I was called to see her when she appeared to be dangerously ill, and immediately applied the wet compress, using ice-water. The wet cloth was several folds in thickness, and covered the entire chest; it was covered with a dry cloth. The relief was almost instantaneous. It has been several years since I first prescribed for this lady. She has had only two or three severe attacks since I first saw her; and it has been very seldom that she has been troubled at all. She says she had never expected to enjoy such health. I believe that she still wears the wet compress. I know that she did wear it, with occasional intermissions for nearly two years. In the asthma cases that I have treated, during the spell, I have always found it necessary to re-wet the cloth as often as it began to get warm; like the application of cold water to a burn, it only affords relief while the water is cold; the pain in the one case, and the difficulty of breathing in the other, return as soon as the water begins to get warm. But between the paroxysms, the wet compress need not be changed, unless it becomes too hot, or gets dry.

To conclude, I would again say, that physicians ought to examine this water-cure system with care. Water is a remedy always at hand, and it is a powerful remedy. Why should we not try it, and use it if we find it valuable?

June, 1852.

